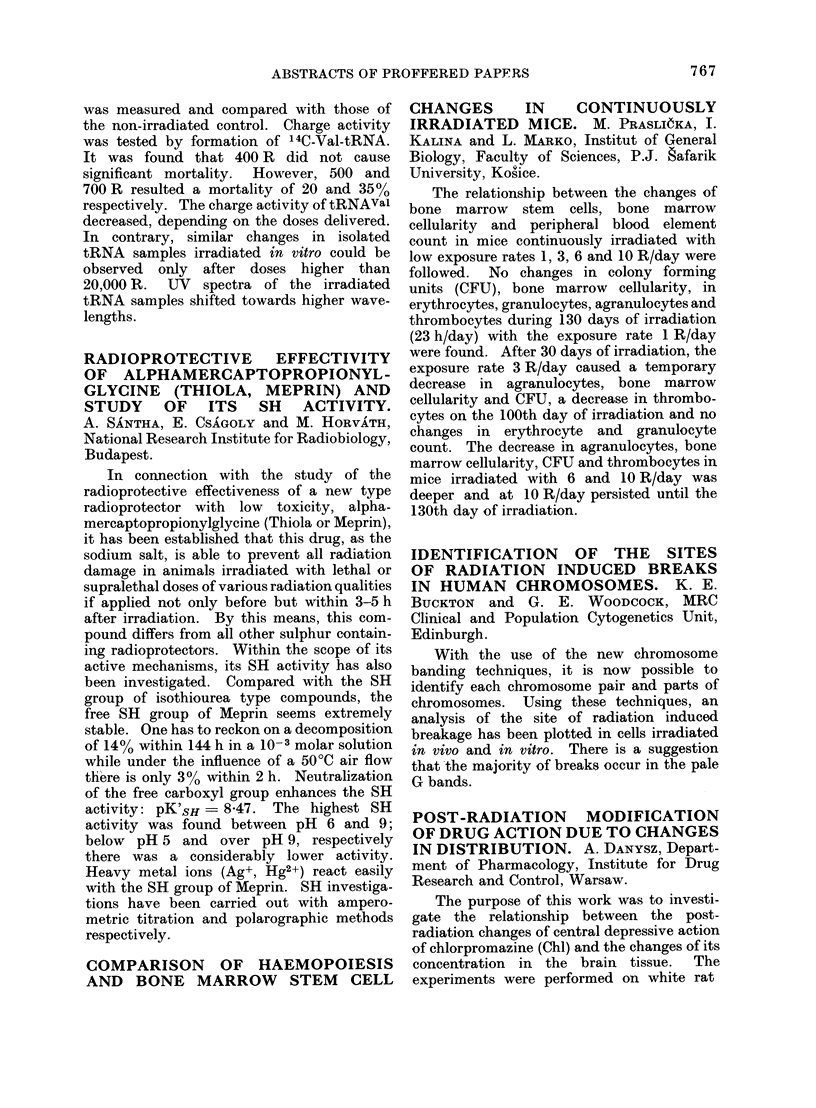# Proceedings: Comparison of haemopoiesis and bone marrow stem cell changes in continuously irradiated mice.

**DOI:** 10.1038/bjc.1975.342

**Published:** 1975-12

**Authors:** M. Praslicka, I. Kalina, L. Marko


### COMPARISON OF HAEMOPOIESIS AND BONE MARROW STEM CELL CHANGES IN CONTINUOUSLY IRRADIATED MICE.

M. Praslička, I. Kalina and L. Marko, Institut of General Biology, Faculty of Sciences, P.J. Šafarik University, Košice.

The relationship between the changes of bone marrow stem cells, bone marrow cellularity and peripheral blood element count in mice continuously irradiated with low exposure rates 1, 3, 6 and 10 R/day were followed. No changes in colony forming units (CFU), bone marrow cellularity, in erythrocytes, granulocytes, agranulocytes and thrombocytes during 130 days of irradiation (23 h/day) with the exposure rate 1 R/day were found. After 30 days of irradiation, the exposure rate 3 R/day caused a temporary decrease in agranulocytes, bone marrow cellularity and CFU, a decrease in thrombocytes on the 100th day of irradiation and no changes in erythrocyte and granulocyte count. The decrease in agranulocytes, bone marrow cellularity, CFU and thrombocytes in mice irradiated with 6 and 10 R/day was deeper and at 10 R/day persisted until the 130th day of irradiation.